# Impact of beam‐hardening corrections on proton relative stopping power estimates from single‐ and dual‐energy CT

**DOI:** 10.1002/acm2.13711

**Published:** 2022-07-11

**Authors:** Michael S. Chacko, Dee Wu, Hardev S. Grewal, Jagadeesh R. Sonnad

**Affiliations:** ^1^ Department of Medical Physics and Dosimetry Oklahoma Proton Center Oklahoma City Oklahoma USA; ^2^ Department of Radiological Sciences University of Oklahoma Health Sciences Center Oklahoma City Oklahoma USA; ^3^ Department of Radiation Oncology University of Florida College of Medicine Gainesville Florida USA; ^4^ Department of Radiation Oncology University of Florida Health Proton Therapy Institute Jacksonville Florida USA

**Keywords:** 3D printing, CT, dual‐energy CT, proton therapy

## Abstract

A major contributing factor to proton range uncertainty is the conversion of computed tomography (CT) Hounsfield units (HU) to proton relative stopping power (RSP). This uncertainty is heightened in the presence of X‐ray beam‐hardening artifact (BHA), which has two manifestations: cupping and streaking, especially in and near bone tissue. This uncertainty can affect the accuracy of proton RSP calculation for treatment planning in proton radiotherapy. Dual‐energy CT (DECT) and iterative beam‐hardening correction (iBHC) both show promise in mitigating CT BHA. This present work attempts to analyze the relative robustness of iBHC and DECT techniques on both manifestations of BHA.

The stoichiometric method for HU to RSP conversion was used for single‐energy CT (SECT) and DECT‐based monochromatic techniques using a tissue substitute phantom. Cupping BHA was simulated by measuring the HU of a bone substitute plug in wax/3D‐printed phantoms of increasing size. Streaking BHA was simulated by placing a solid water plug between two bone plugs in a wax phantom. Finally, the effect of varying calibration phantom size on RSP was calculated in an anthropomorphic head phantom.

The RSP decreased −0.002 cm^–1^ as phantom size increased for SECT but remained largely constant when iBHC applied or with DECT techniques. The RSP varied a maximum of 2.60% in the presence of streaking BHA in SECT but was reduced to 1.40% with iBHC. For DECT techniques, the maximum difference was 2.40%, reduced to 0.6% with iBHC. Comparing calibration phantoms of 20‐ and 33‐cm diameter, maximum voxel differences of 5 mm in the water‐equivalent thickness were observed in the skull but reduced to 1.3 mm with iBHC. The DECT techniques excelled in mitigating cupping BHA, but streaking BHA still could be observed. The use of iBHC reduced RSP variation with BHA in both SECT and DECT techniques.

## 1 INTRODUCTION

The benefits of the increasingly conformal dose distributions of proton radiotherapy can only be realized through a careful consideration of the uncertainties resulting from the use of computed tomography (CT) Hounsfield units (HU) to calculate compositional parameters of tissue in the underlying dose computation.^1^ These parameters in practice are typically the proton relative stopping power (RSP) or mass density (*ρ*) of tissue derived from a user‐generated single‐energy CT (SECT) HU calibration curve.^2^, ^3^ The associated uncertainty of such calibrations may be as high as 3.5%–8% of the proton range, owing in large part to variations in HU and tissue composition.^4^, ^5^, ^6^, ^7^ The use of dual‐energy CT (DECT) has promise in reducing these uncertainties. By extracting the compositional effective atomic number (EAN) and relative‐to‐water electron density (RED or *ρ_e_*) as proposed by Rutherford et al. and Alvarez and Macovski, DECT techniques can reportedly achieve reductions in tissue composition uncertainty.^8^, ^9^, ^10^ The use of DECT has also been reported to reduce proton RSP error to between 0.5% and 1.5%.^11^ However, in the specific case of HU variation, especially in the presence of CT beam hardening, the benefit from the use of DECT has not been well characterized and warrant further investigation.

The reasons for HU variation can be multifaceted, but components are related to CT beam‐hardening artifact (BHA), X‐ray tube heating, and position in the scan volume among other factors. CT beam hardening is the deviation from a linear relationship between measured X‐ray projections and the X‐ray path length due to selective absorption of the low‐energy portion of the polychromatic X‐ray spectrum and associated shift in the mean attenuation coefficient.^12^ It has two distinct manifestations: cupping and streaking. Cupping is a low‐frequency CT shift, commonly seen in a uniform cylindrical phantom as the difference between the HU at the center and the periphery. Streaking is a high‐frequency CT shift, manifesting as dark bands connecting dense material or tissue. It is important to note that linearization correction methods for water and soft tissue can correct for cupping, but not streaking, commonly caused by bone in the patient.^13^ More sophisticated beam‐hardening correction uses software to characterize tissues as mixtures of water‐ or bone‐like tissues iteratively and applies respective corrections in either the image or projection space, such as the Siemens iterative beam‐hardening correction (iBHC) algorithm (Siemens Healthineers, Erlangen, Germany); these methods can address both manifestations of BHA.^14^, ^15^, ^16^, ^17^, ^18^, ^19^

**FIGURE 1:**
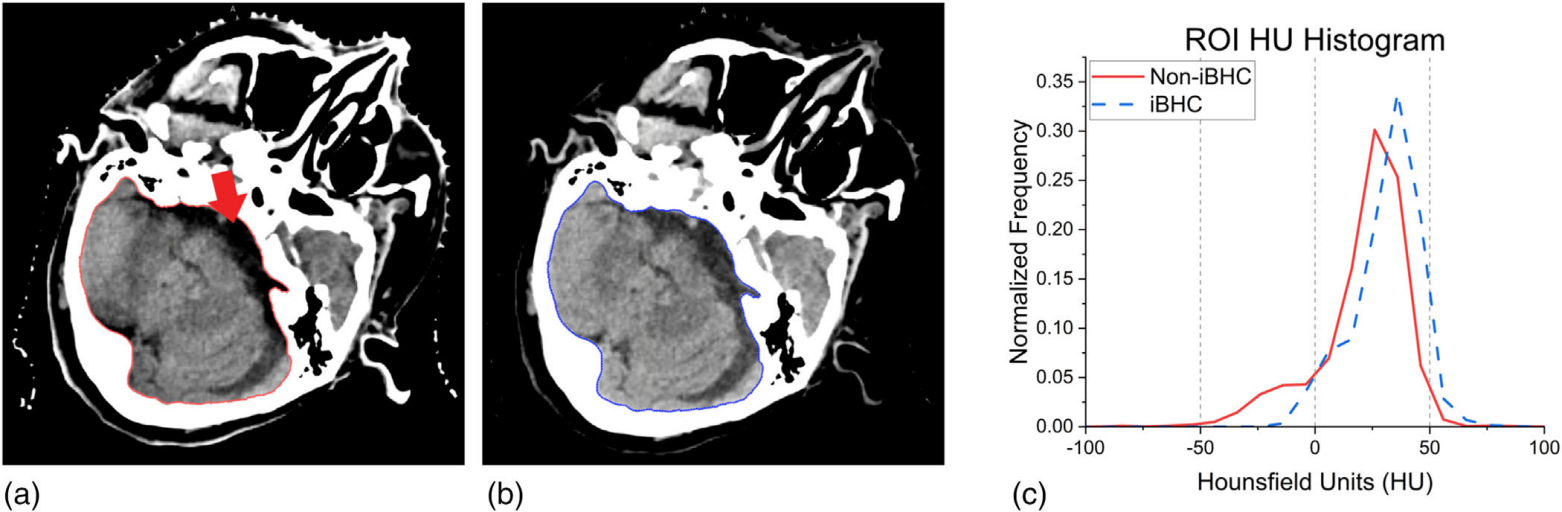
Clinical example of beam‐hardening artifact (BHA) uncorrected in (a) and with iterative beam‐hardening correction (iBHC) applied (b). Difference in extracted Hounsfield units (HU) of the brain and CSF (red arrow) HU shown in the histogram (c) with notable low‐HU tail for the uncorrected scan

The use of DECT has potential to correct for BHA robustly.^20^ By leveraging data obtained with two spectra, DECT can be used to approximate reconstruction at single energy. Purely monochromatic CT reconstructed images contain no BHA due to the lack of any spectral shift. However, many DECT techniques can only approximate these mono‐energies due to reliance on polychromatic acquisitions as a basis for the generation of pseudo‐monochromatic reconstructions. Therefore, techniques such as Siemens’ DECT‐derived “Mono” and “Mono+” algorithms cannot completely remove artifacts such as BHA.^21^ Michalak et al. characterized HU stability of these specific algorithms across increasing phantom sizes for various energies and techniques and found marked improvement over SECT in the context of proton RSP.^22^ These algorithms generate pseudo‐monochromatic reconstructions using a linear combination of the constituent SECT scans. Wohlfahrt et al. also found these benefits for the same algorithms in the proton RSP computation, with a maximal beam‐hardening reduction of 23% at 79 keV in comparison with a 120‐kVp SECT reference technique.^23^ However, these experimental setups did not induce the high‐frequency streaking BHA that affects tissues adjacent to dense bone, such as brain medial to the petrous pyramids of the skull (Figure 1). Yu et al. found significant streaking BHA in pseudo‐monochromatic images generated by a projection‐space technique in a phantom at energies less than 100 keV.^24^ Ueguchi et al. described the variability of image‐based pseudo‐monochromatic CT numbers on constituent SECT images “that have already undergone beam hardening”.^25^ It follows then that the robustness of DECT and algorithmic beam‐hardening corrections on both manifestations of BHA needs to be examined.

This present work attempts to analyze the relative robustness of algorithmic beam‐hardening corrections such as iBHC‐ and DECT‐based techniques when applied to low‐ and high‐frequency components of BHA as they relate to HU to RSP conversions. We also examine the impact of beam hardening on the HU to RSP calibration by examining water‐equivalent thickness (WET) estimates of an anthropomorphic head phantom at a clinical proton therapy energy.

## 2 METHODS AND MATERIALS

Measurements were conducted with a Siemens SOMATOM CONFIDENCE RT Pro CT scanner equipped with Siemens *syngo*.via software (Siemens Healthineers, Erlangen, Germany). The CT scanner is capable of reconstructing pseudo‐monochromatic images from dual‐spiral DECT and can provide direct estimates of the EAN (*Z*_eff_) and RED (*ρ_e_*) through the Rho/Z algorithm. Software‐based beam‐hardening correction was achieved using Siemens iBHC algorithm with the “bone” setting with the standard or default cupping correction. Phantoms were machined from Freeman Blue Machinable Wax (Freeman Manufacturing & Supply Company, Avon, Ohio) that is used for patient‐specific compensators in our clinical practice.

Three SECT spectra of 80, 120, and 140 kVp and three pseudo‐monochromatic reconstructions corresponding to 60, 80, and 100 keV as well as *syngo*.via Rho/Z reconstructions were used in this study for all sections unless otherwise indicated. The reported CT numbers from the Mono+ reconstruction algorithm are not directly comparable to corresponding monochromatic X‐rays of the same nominal energy due to vendor and scanner‐specific weighting during reconstruction. The energy selection was motivated by maximizing beam‐hardening reduction; Wohlfahrt et al. reported a maximum beam‐hardening reduction factor at 79 keV with the Mono+ algorithm in a particular experimental setup.^23^ The pseudo‐monochromatic images and Rho/Z estimates were generated from a DECT energy pair of 80/140 kVp scanned sequentially. Each technique was calibrated and measured with and without iBHC‐bone selected for reconstruction.

Scan techniques utilized are summarized in Table 1. No intra‐scan mAs modulation was utilized, fixed mAs was determined by CTDI_vol_ matching between SECT and DECT techniques. Constituent DECT scans were equally weighted for CTDI_vol_ summation. All pseudo‐monochromatic reconstructions were generated using the Mono+ algorithm using the 80/140‐kVp energy pair.

**TABLE 1:** Scan techniques utilized in this work

Scan techniques	
Energy (kVp)	80, 120, 140
mAs	Scaled with CTDIvol
CTDI_vol_ (mGy)	28.2
Reconstruction kernel	Qr40/Mono+
Scan FOV (cm)	50
Slice thickness (mm)	1
Collimation	20 × 0.6 mm

*Note*: Pseudo‐monochromatic reconstructions were generated from the 80/140‐kVp energy pair using the Mono+ algorithm.

The region‐of‐interest (ROI) segmentation for all studies was performed with the RayStation 9B (RaySearch Laboratories, Stockholm, Sweden) treatment planning system (TPS), and HU histogram extraction and analysis with DICOM tools in Matlab 2021b (The Mathworks Inc., Natick, MA). The ROIs for tissue substitute plugs were contoured and contracted 2 mm uniformly to avoid values at the plug and phantom interface and to maintain a consistent size across all samples.

### 2.1 CT calibration

**FIGURE 2:**
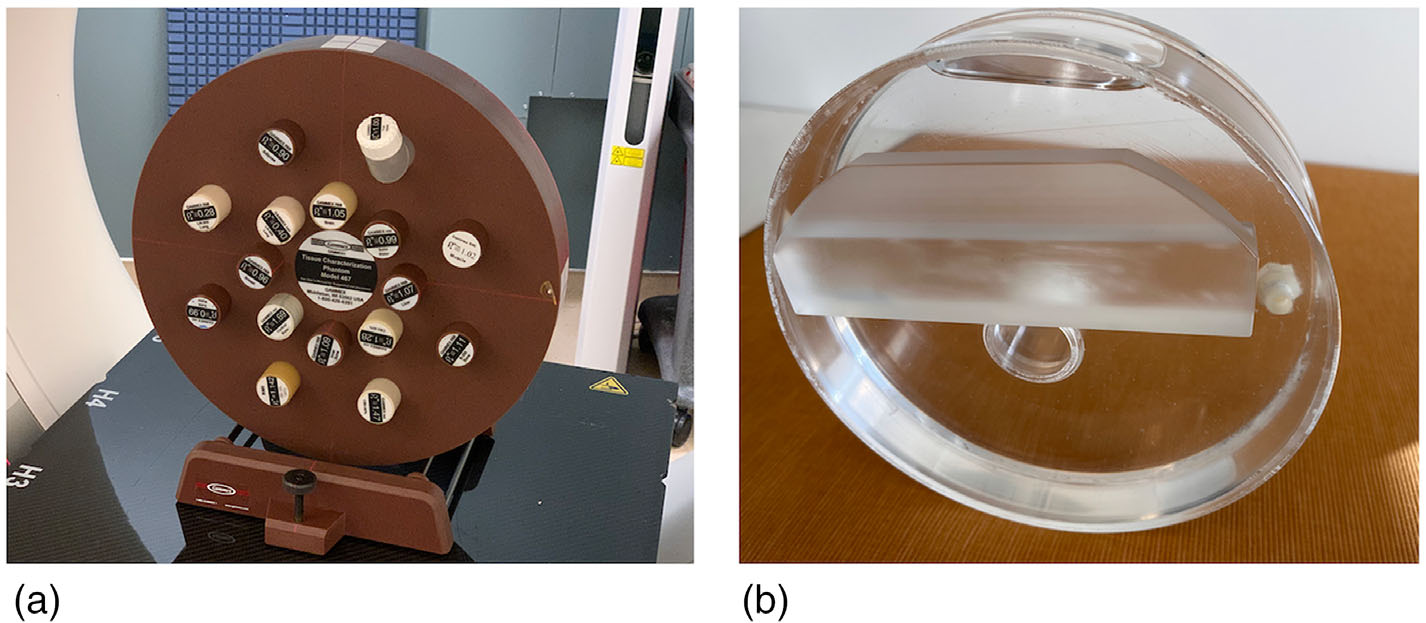
Computed tomography (CT) calibration phantoms used in this study. Model 467 phantom of 33‐cm diameter with tissue substitute plugs (a). Custom water‐filled PMMA phantom of 20 cm diameter (b)

Calibration of each SECT and DECT pseudo‐monochromatic technique–utilized tissue calibration plugs of an electron density phantom (Tissue Characterization Phantom Model 467, Sun Nuclear Corporation) summarized in Table 2 scanned with the Qr40 kernel with no mAs modulation at 1.0‐mm slice thickness in a 20 × 0.6‐mm collimation with 50‐cm field of view (FOV). These plugs were used with the supplied phantom and a custom polymethyl methacrylate (PMMA) phantom filled with water of 20 cm in diameter (Figure 2) to generate two distinct calibration curves. The Rho/Z technique from the *syngo*.via software does not require user calibration to generate EAN and RED estimates.

**TABLE 2:** Tissue substitute plugs used for calibration

Calibration plug	*ρ_e_*	*Z*_eff_	Δ*Z*_eff_
LN‐300 lung	0.276	7.55	0.03
LN‐450 lung	0.432	7.52	0.03
AP6 adipose	0.924	6.17	0.02
BR‐12 breast	0.96	6.87	0.03
Water insert	1	7.45	0.02
CT solid water	0.99	7.66	0.03
BRN‐SR2 brain	1.049	6.04	0.03
LV1 liver	1.062	7.66	0.03
IB inner bone	1.082	10.28	0.03
B200 bone mineral	1.099	10.29	0.03
CB2‐30% CaCO_3_	1.279	10.76	0.02
CB2‐50% CaCO_3_	1.471	12.4	0.01
SB3 cortical bone	1.693	13.51	0.01

*Note*: Values for RED were obtained from the vendor and are mix specific. Values for EAN and associated uncertainty taken from Bourque et al.

**^Abbreviations: CT,^ :**computed tomography; EAN, effective atomic number; RED, relative‐to‐water electron density.

The stoichiometric calibration method of Schneider et al. as adapted by Bourque et al. was utilized, in which the model HU (*U*_model_) response is a product of RED and some function of EAN:

(1) $$\;U_{\mathrm{model}}=\frac{\left({\mathrm{HU}}_{\mathrm{model}}+1000\right)}{1000}\;=\rho_{e}\;f\left(Z_{\mathrm{eff}}\right)$$

where the function of EAN is fit as a power series in *Z*_eff_ through calibration:

(2) $$f\left(Z_{\mathrm{eff}}\right)=\;\sum_{m\;=\;1}^{M}{\hat{b}}_{m}Z_{\mathrm{eff}}^{M-1}$$

to obtain fitting coefficients ${\hat{b}}_{m}$ during calibration for each energy spectrum using least‐squares fitting through the SciPy Python library.^26^, ^27^ The HU of each tissue substitute plug was read using a 4.0 cm^2^ ROI along the length of the tissue plug. The degree of polynomial fit of (M−1)=5 was chosen based on residual analysis of the model HU response consistent with the implementation of Xie et al.^28^ The model HU response was obtained for 33 biological tissues of known elemental composition from ICRP 23 and plotted against calculated proton RSP at 200 MeV using the Bethe–Bloch formula:

(3) $$\begin{array}{ccc} & & \mathrm{RSP}\;=\;\frac{S}{S_{\mathrm{water},\;200\;\mathrm{MeV}}},\;\\ & & S\;=\;\rho_{e}\frac{k_{0}z^{2}}{\beta^{2}}\left[\ln\left(\frac{2m_{e}c^{2}\beta^{2}}{I\left(1-\beta^{2}\right)}\right)-\beta^{2}\right]\; \end{array}$$

where $k_{0}\;=\;\;0.17045\;\;\mathrm{MeV}/\mathrm{cm},$ z=1, $m_{e}\;c^{2}\;=\;\;0.511\;\;\mathrm{MeV}$, β=0.5662c at 200 MeV, and *I* is the mean excitation value.^29^ The resulting calibration curve may then be used to obtain the derived RSP for any measured HU.

### 2.2 Cupping artifact

**FIGURE 3:**
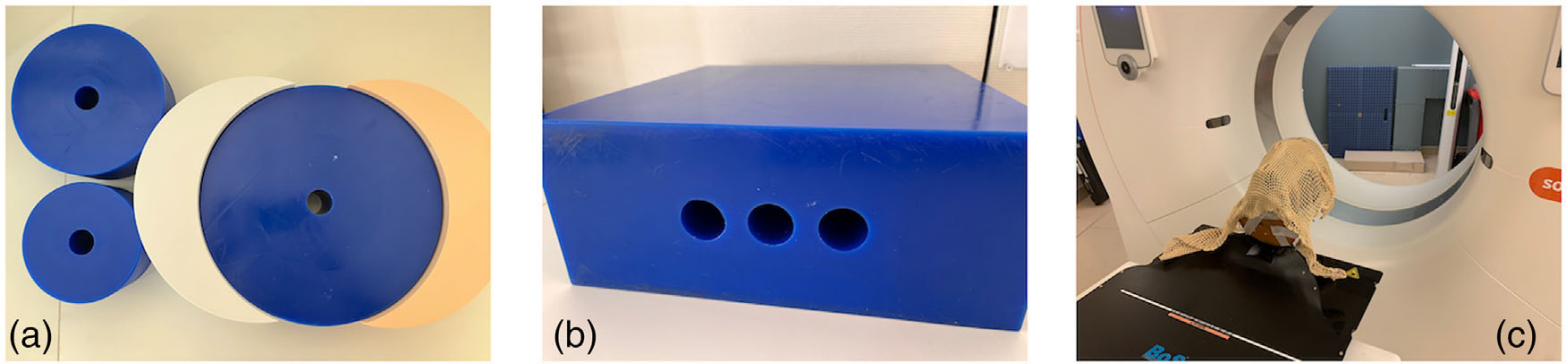
Phantom scenarios and setups: cupping beam‐hardening artifact (BHA) with three wax phantoms and 3D‐printed extensions (a), three‐hole streaking BHA wax phantom (b), and anthropomorphic head phantom (c)

In order to examine the cupping component of BHA, the mean HU of the cortical bone tissue plug was measured in phantoms of increasing size as determined by the effective diameter (*d*_eff_):

(4) $$d_{\mathrm{eff}}\left(\mathrm{cm}\right)\;=\;\sqrt{\mathrm{AP}\times \mathrm{LAT}}$$

where AP and LAT are the anterior–posterior and lateral dimensions of the phantom, respectively.^30^ Wax cylindrical phantoms of 7‐cm length and with diameters of 13.5, 17.8, and 26.9 cm were constructed with a 28‐mm diameter hole to fit the cortical bone tissue plug. The largest phantom was extended with lateral extensions, resulting in a *d*_eff_ of 30.2 cm (Figure 3a). Extensions to the wax phantom were 3D‐printed from polylactic acid at 84% infill density to match the mean HU of the wax using Ultimaker Cura software with an Ultimaker S5 printer (Ultimaker B.V., Utrecht, Netherlands). All phantoms were scanned with each technique with and without iBHC applied. Three repeat scans for each technique were taken to verify reproducibility.

### 2.3 Streaking artifact

**FIGURE 4:**
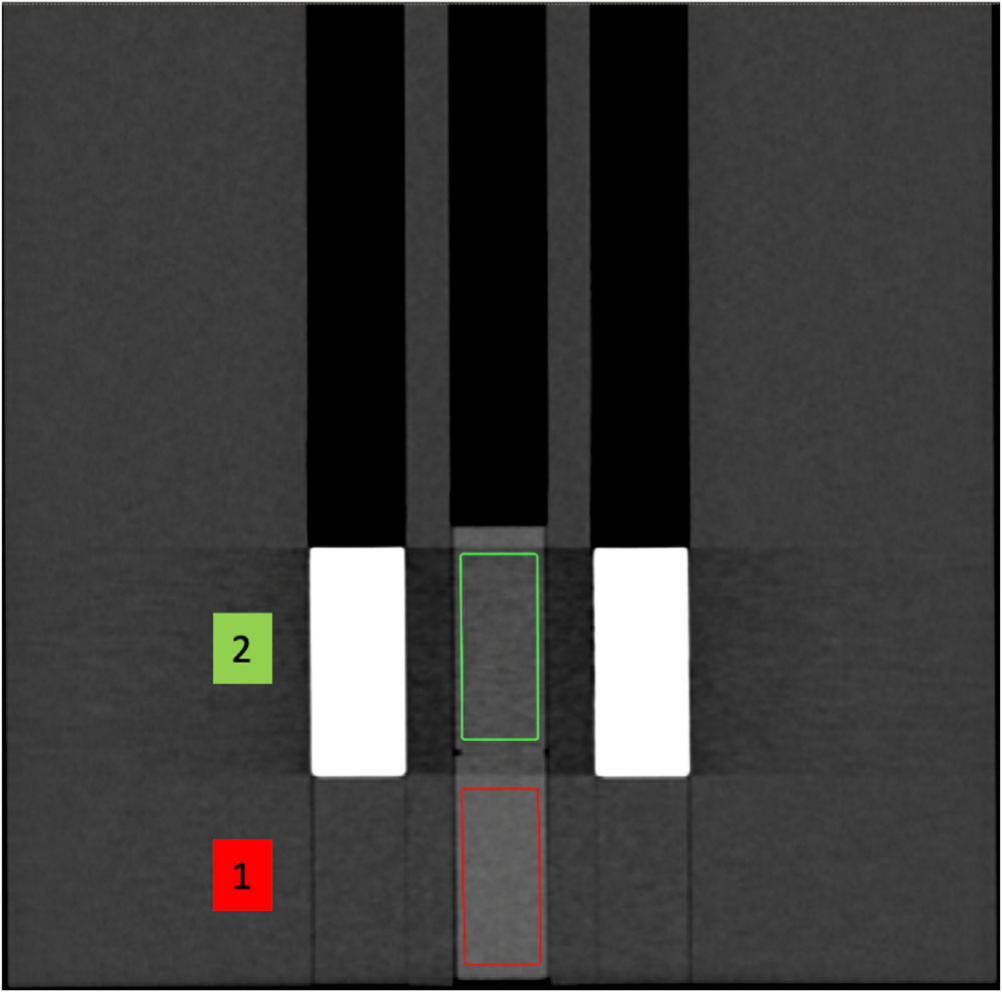
Hounsfield unit (HU) extraction regions of phantom shown in Figure 3b. Region of interest (ROI) extracted from identical solid water plugs in each region. Region 1 simulates the artifact‐free condition and region 2 with induced streaking beam‐hardening artifact (BHA) between two identical cortical bone plugs.

To induce the streaking component of BHA, the mean HUs of two solid water plugs were measured in a custom‐designed wax phantom (Figure 3b). The phantom measured 330 × 330 × 120 mm (*L* × *W* × *H*) with three holes throughout the length of 28‐mm diameter and were 15 mm apart (edge‐to‐edge) to fit the tissue and wax plugs. Measurements were conducted in two distinct regions (Figure 4) to assess the difference of the mean HU of the solid water plugs between them. Region 1 simulated the artifact‐free scenario, with wax plugs in the holes surrounding the central hole, filled with a solid water plug. Region 2 induced the streaking BHA using two cortical bone plugs surrounding a solid water plug in the center. All phantoms were scanned with each technique with and without iBHC applied. Three repeat scans for each technique were taken for error analysis. Segmentation in the TPS resulted in a 26‐mm axial diameter ROI along the length of the plug for both regions with a 68‐mm length.

### 2.4 Anthropomorphic phantom measurement

To estimate the impact of the CT calibration phantom size on proton RSP for each CT technique, an anthropomorphic head phantom (Alderson RANDO, Radiology Support Devices Inc., Long Beach, CA) was with and without iBHC applied (Figure 3c). For each technique, the HU were converted pixel‐wise to RSP using both the Model 467 phantom and the smaller acrylic phantom generated HU to RSP calibrations. Both phantom calibrations were used to generate RSP maps of the RANDO head phantom. These maps were converted to WET using the corresponding voxel size and summed along a 90‐degree projection using Matlab. The difference in the WET projections (ΔWET) was generated for comparison.

## 3 RESULTS

### 3.1 Calibration

**FIGURE 5:**
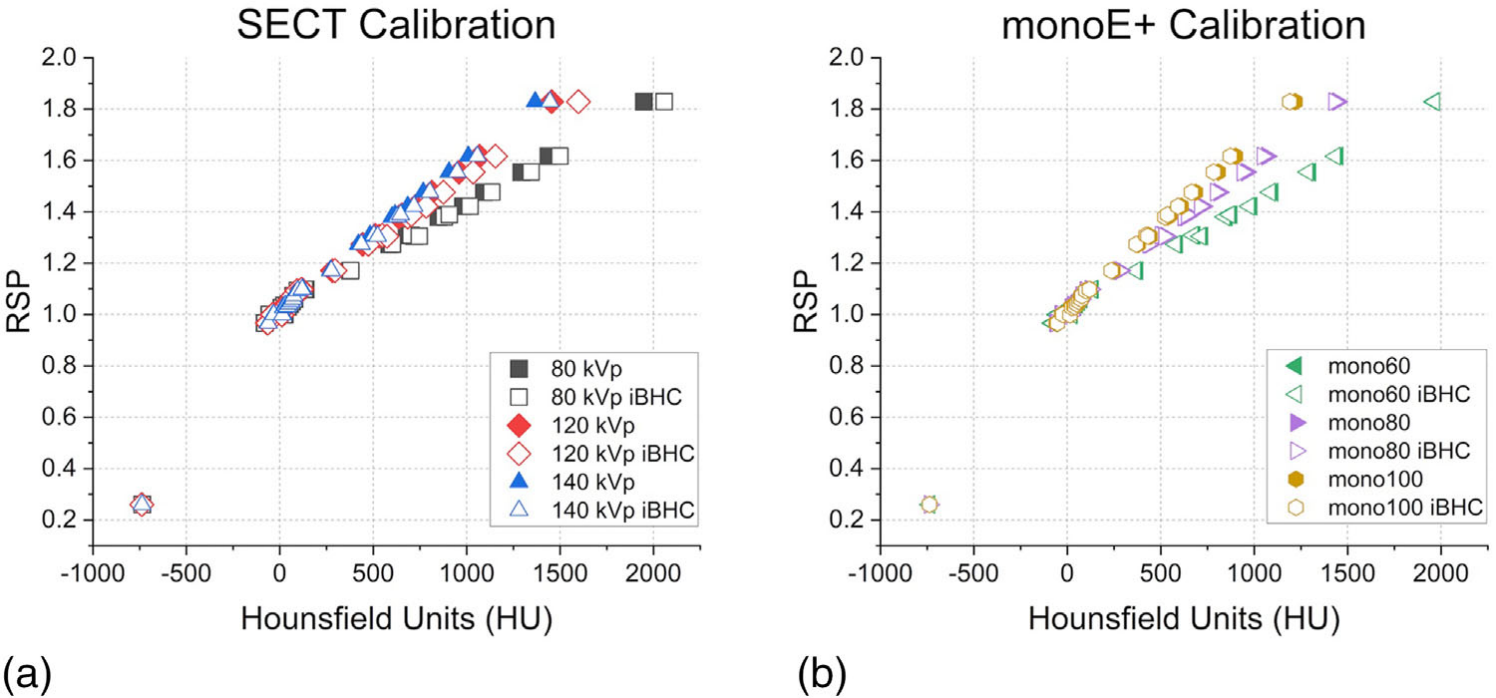
Hounsfield units (HU) to relative stopping power (RSP) calibration results for single‐energy computed tomography (SECT) (a) and Mono+ (b) techniques with and without iterative beam‐hardening correction (iBHC) applied

Figure 5a shows the variation of the computed RSP with HU for 33 human tissues as characterized in ICRP 23 for 80, 120, and 140‐kVp SECT calibrations with and without the inclusion of iBHC bone correction. In general, the calibration curve slope increases as energy increases for HU values greater than approximately 200 HU. The HU differences between each base technique and its respective iBHC included version at each given RSP generally decreased as the spectrum energy increased, with maximal differences of approximately 100 HU at 80 kVp for bone. Figure 5b shows the DECT‐derived pseudo‐monochromatic technique calibrations with associated iBHC curves; the HU differences between each technique and its iBHC version were smaller than SECT techniques, with a maximum difference of approximately 30 HU at 60 keV for bone.

### 3.2 Cupping BHA

**FIGURE 6:**
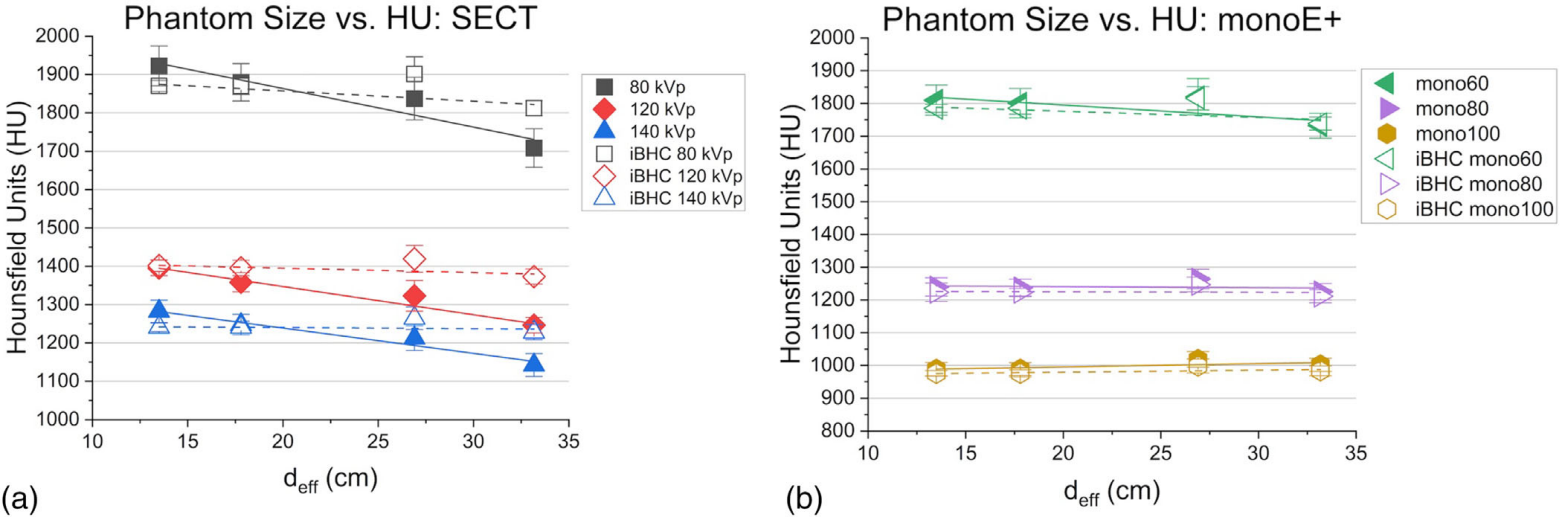
Phantom‐effective diameter versus cortical bone Hounsfield units (HU) for single‐energy computed tomography (SECT) (a) and dual‐energy computed tomography (DECT) Mono+ (b) techniques with and without iterative beam‐hardening correction (iBHC) applied. Lines indicate linear fits for standard (solid) and iBHC techniques (dashed). Error bars include HU variation within the region of interest (ROI).

The cortical bone plug HU value is plotted in Figure 6a as a function of the *d*_eff_ for the SECT techniques. As the phantom size (*d*_eff_) increased, the mean HU decreased for techniques without iBHC to a maximum difference of approximately −200 HU at 80 kVp and Pearson's correlation coefficient (*r*) <−0.95 for all techniques indicating a strong negative relationship. The slope of the linear fit of *d*_eff_ and HU decreased from −9.9 to −6.6 HU/cm from 80 to 140 kVp. In contrast, the SECT techniques with iBHC indicated a weaker correlation of the mean HU from the *d*_eff_ of the phantom, with the strongest Pearson's correlation coefficient of *r* = −0.48 and slope of −2.7 HU/cm at 80 kVp.

Figure 6b shows the cortical bone plug HU values for the DECT‐derived pseudo‐monochromatic techniques as phantom *d*_eff_ increased. A weak correlation of the mean HU as a function of the phantom size was indicated with a correlation coefficient of *r* = −0.7 and slope of −3.6 HU/cm at 60 keV, and slopes of −0.3 HU/cm at 80 keV and +1 HU/cm for 100 keV. Minimal differences were obtained with iBHC applied.

**FIGURE 7:**
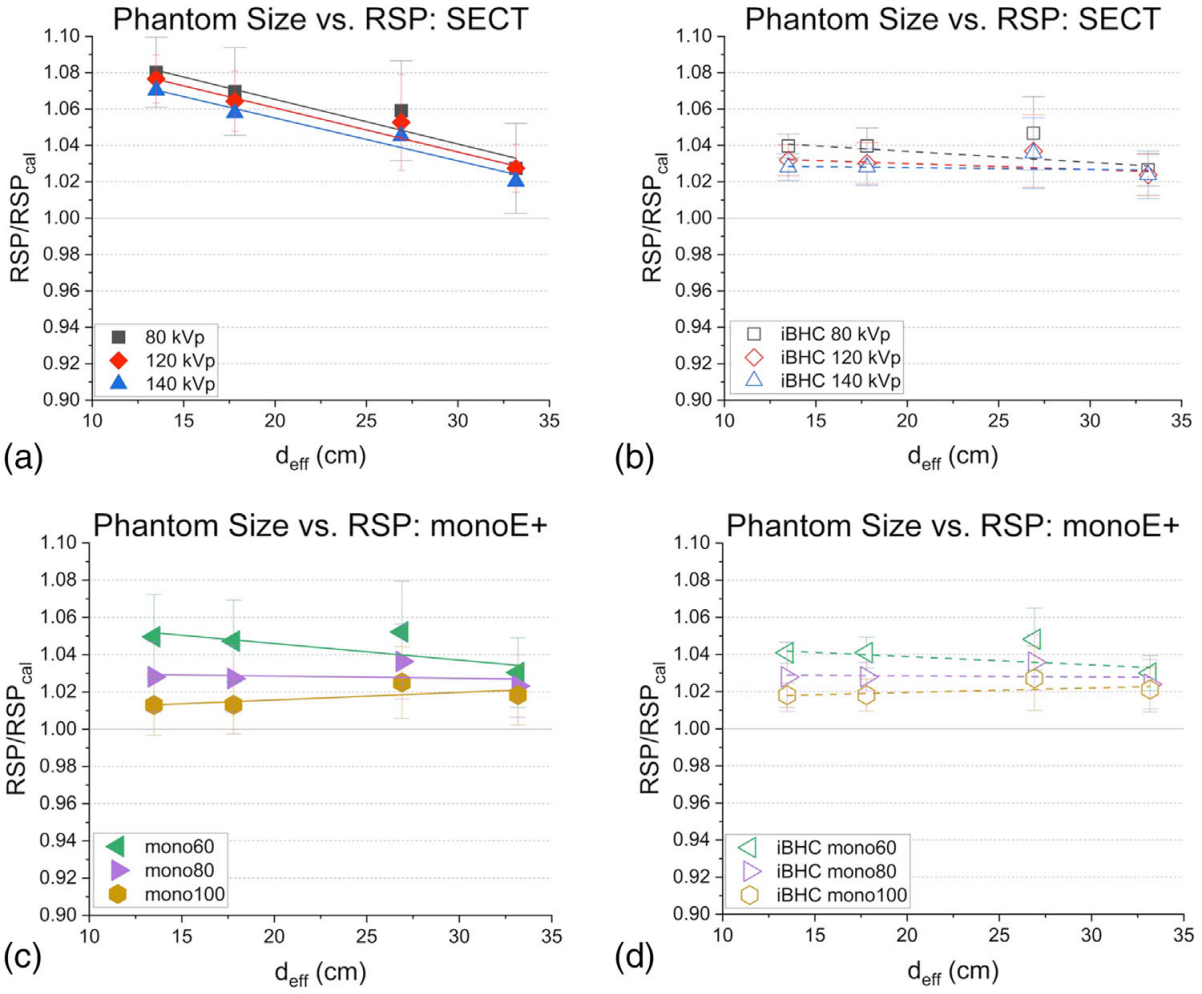
Phantom effective diameter versus cortical bone relative stopping power (RSP) for single‐energy computed tomography (SECT) (a) SECT with iterative beam‐hardening correction (iBHC) (b) and dual‐energy computed tomography (DECT)‐derived Mono+ (c) with iBHC (d). Lines indicate associated linear fits. RSP_cal_ is the calibration condition. Error bars include RSP variation within the region of interest (ROI) and calibration model uncertainty.

Each technique's respective calibration was used to interpolate the RSP from the mean HU values of Figure 6 and was then normalized to that of the cortical bone plug RSP value during calibration with the Model 467 phantom (Figure 7). Slopes of the *d*_eff_ to RSP ratio relation were similar for all SECT energies with −0.003, −0.002, and −0.002 cm^–1^ for 80, 120, and 140 kVp. The SECT techniques with iBHC applied reduced these slopes by a factor of approximately 2 or greater. The pseudo‐monochromatic techniques exhibited a much weaker dependency of the RSP ratio on *d*_eff_ with slopes <−0.001 cm^–1^ for all techniques and with iBHC applied.

**FIGURE 8:**
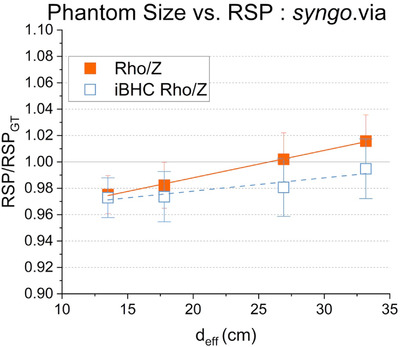
Phantom‐effective diameter versus cortical bone relative stopping power (RSP) for the *syngo*.via Rho/Z technique with and without iterative beam‐hardening correction (iBHC) applied. Lines indicate linear fits for Rho/Z (solid) and iBHC Rho/Z (dashed). RSP was normalized to that of the calibration condition in the Model 467 phantom. Error bars represent parameter variation within the selected region of interest (ROI).

The ratio of calculated RSP from the EAN and RED reconstructions of the *syngo*.via Rho/Z algorithm to that of ground truth is plotted as a function of *d*_eff_ in Figure 8. A positive correlation can be seen (*r* = 0.99, slope = 0.002 cm^–1^) with an approximate 4% increase in the RSP ratio from the smallest to largest phantom. This difference was reduced to 2% with the addition of iBHC (*r* = 0.94, slope = 0.001 cm^–1^).

### 3.3 Streaking BHA

**FIGURE 9:**
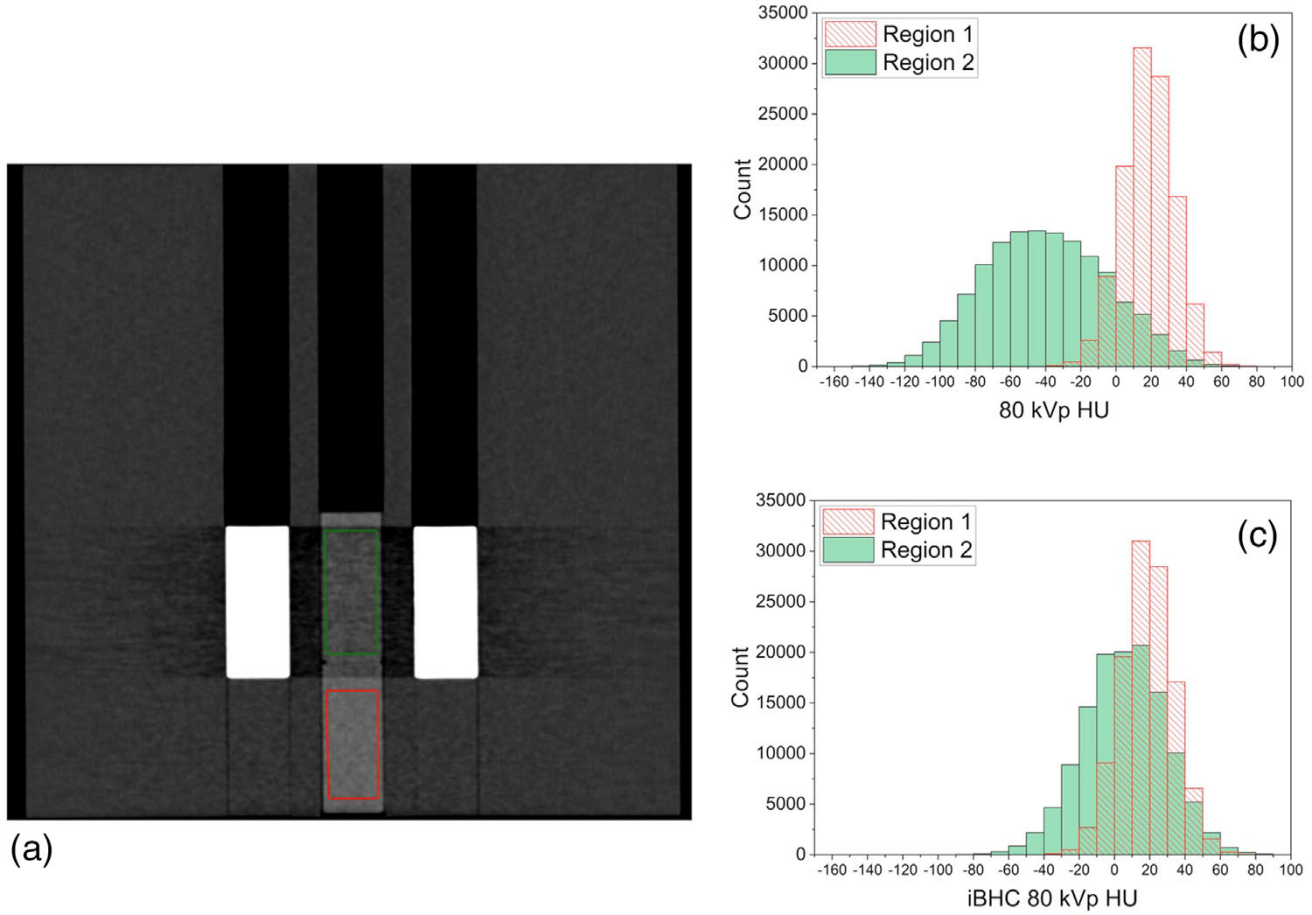
Example histogram analysis at 80 kVp for streaking beam‐hardening artifact (BHA) in phantom (a) without iterative beam‐hardening correction (iBHC) applied (b) and with iBHC applied (c)

**FIGURE 10:**
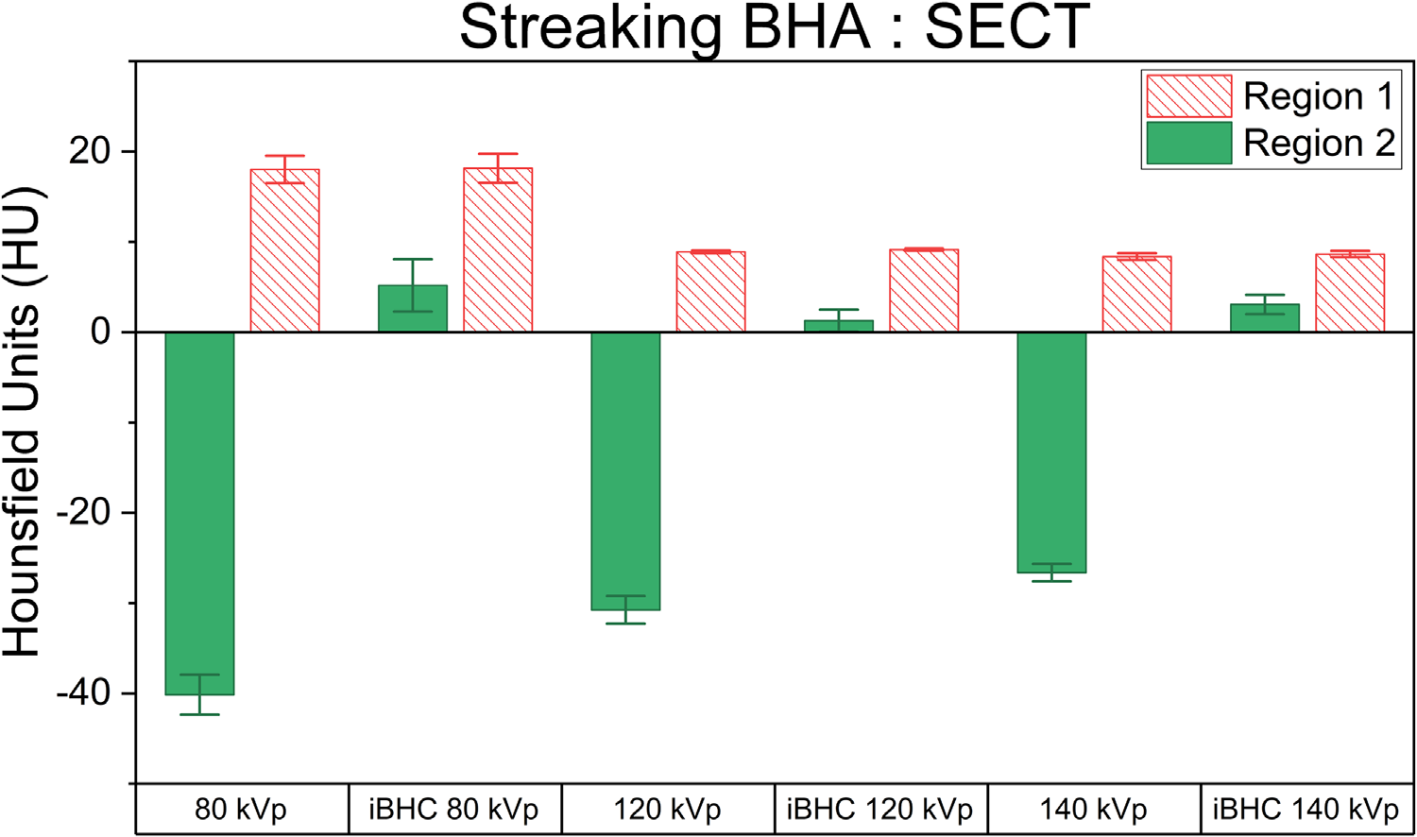
Hounsfield units (HU) differences between artifact‐free region 1 and beam‐hardening artifact (BHA) region 2 for single‐energy computed tomography (SECT) techniques with and without iterative beam‐hardening correction (iBHC) applied. Error bars represent a 95% confidence interval of three scan repeats.

**FIGURE 11:**
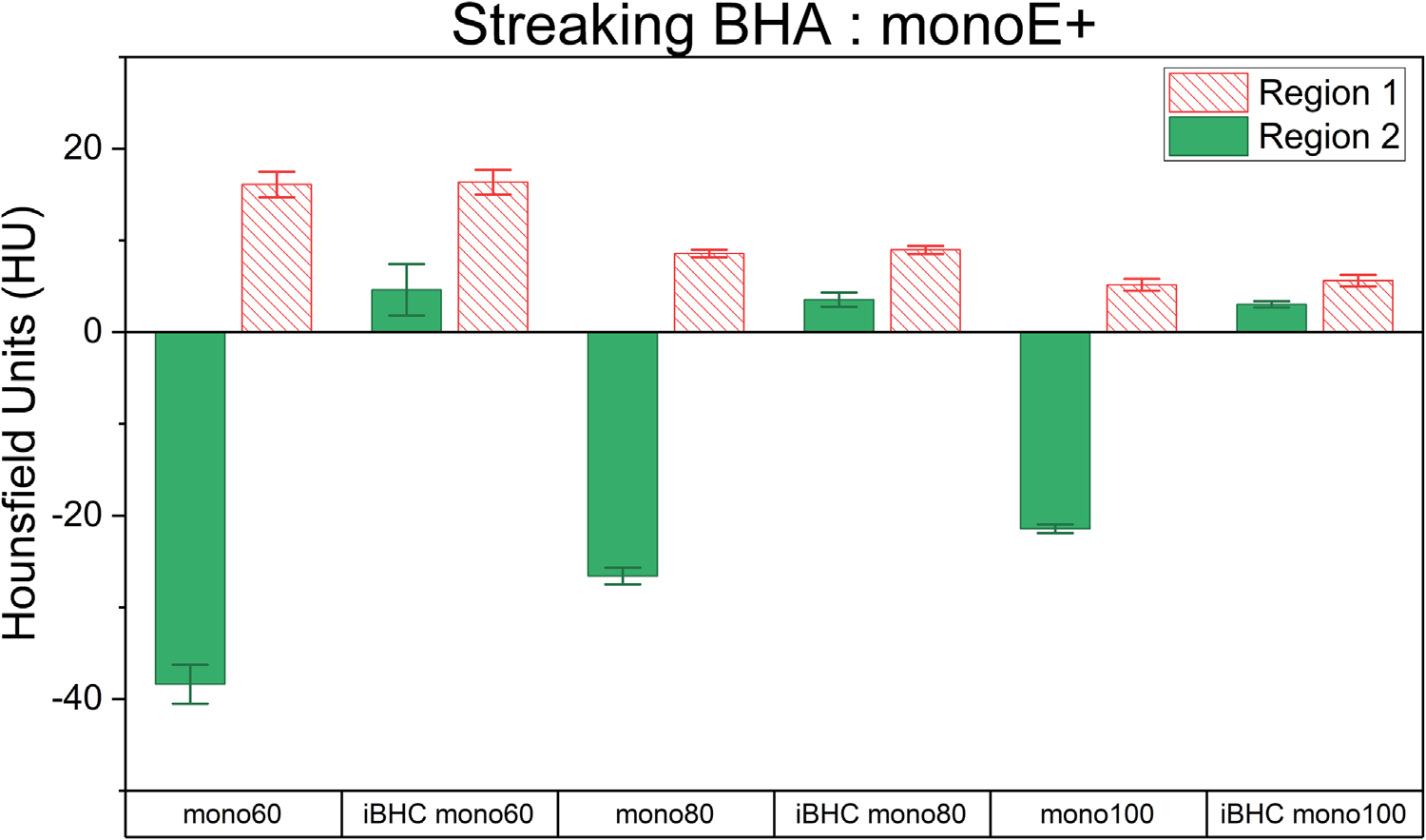
Hounsfield unit (HU) differences between artifact‐free region 1 and beam‐hardening artifact (BHA) region 2 for dual‐energy computed tomography (DECT)‐derived Mono+ techniques with and without iBHC applied. Error bars represent 95% confidence interval of three scan repeats.

The streaking artifact was induced by using two cortical bone plugs lateral to a central solid water plug ROI and compared to the homogeneous region 1 (Figure 4). Example HU distributions for each region at 80 kVp are shown in Figure 9. The mean HU and associated error of identical ROIs for all techniques were analyzed for the SECT techniques (Figure 10) and for the pseudo‐monochromatic reconstructions (Figure 11). The absolute difference in the mean HU (ΔHU) of the ROI between the artifact‐free (region 1) and BHA region (region 2) decreased as the energy increased with ΔHU of 58.2, 39.7, and 35.0 HU for 80, 120, and 140 kVp respectively. The use of iBHC with the SECT techniques reduced the respective ΔHU to 13.0, 7.9, and 5.6 HU. Because the standard deviation of the HU values within the ROI was influenced by the streaking BHA, the error associated with repeatability was assessed using the values obtained by three repeat scans for each technique.

For the DECT pseudo‐monochromatic reconstructions, a similar trend was observed as with SECT. As the reconstructed energy increased, the ΔHU was 54.5, 35.2, and 26.6 HU for 60, 80, and 100 keV, respectively. The inclusion of iBHC reduced ΔHU to 11.7, 5.4, and 2.6 HU with increasing energy.

**FIGURE 12:**
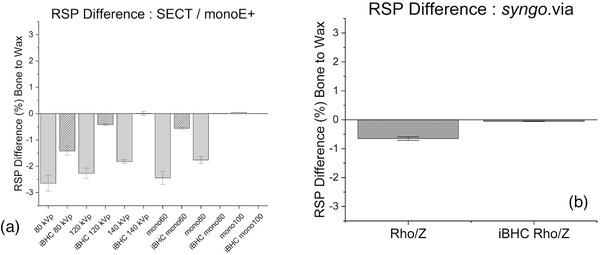
Relative stopping power (RSP) differences between artifact‐free region 1 and beam‐hardening artifact (BHA) region 2 for single‐energy computed tomography (SECT) and dual‐energy computed tomography (DECT)‐derived Mono+ (a) and Rho/Z (b) techniques with and without iterative beam‐hardening correction (iBHC) applied. Error bars represent 95% confidence interval of three scan repeats.

The RSP percent difference was obtained from the respective calibration for each technique (Figure 12a). The absolute difference of the mean RSP (region 1 − region 2) decreased from 2.60% to 1.80% from 80 to 140 kVp for SECT techniques. Similarly, for DECT‐derived Mono+ techniques, they were 2.40%, 1.80%, and <0.05% for 60, 80, and 100 keV, respectively. With the inclusion of iBHC with SECT, these absolute RSP differences reduced to 1.40%, 0.40%, and <0.05% as energy increased. For DECT‐derived monochromatic reconstructions with iBHC, the absolute RSP differences were 0.6%, <0.05%, and <0.05% as reconstructed energy increased.

With the *syngo*.via Rho/Z computed RSP, absolute differences between the regions were 0.6% and 0.05% for Rho/Z and Rho/Z iBHC, respectively (Figure 12b).

### 3.4 Anthropomorphic phantom measurements

**FIGURE 13:**
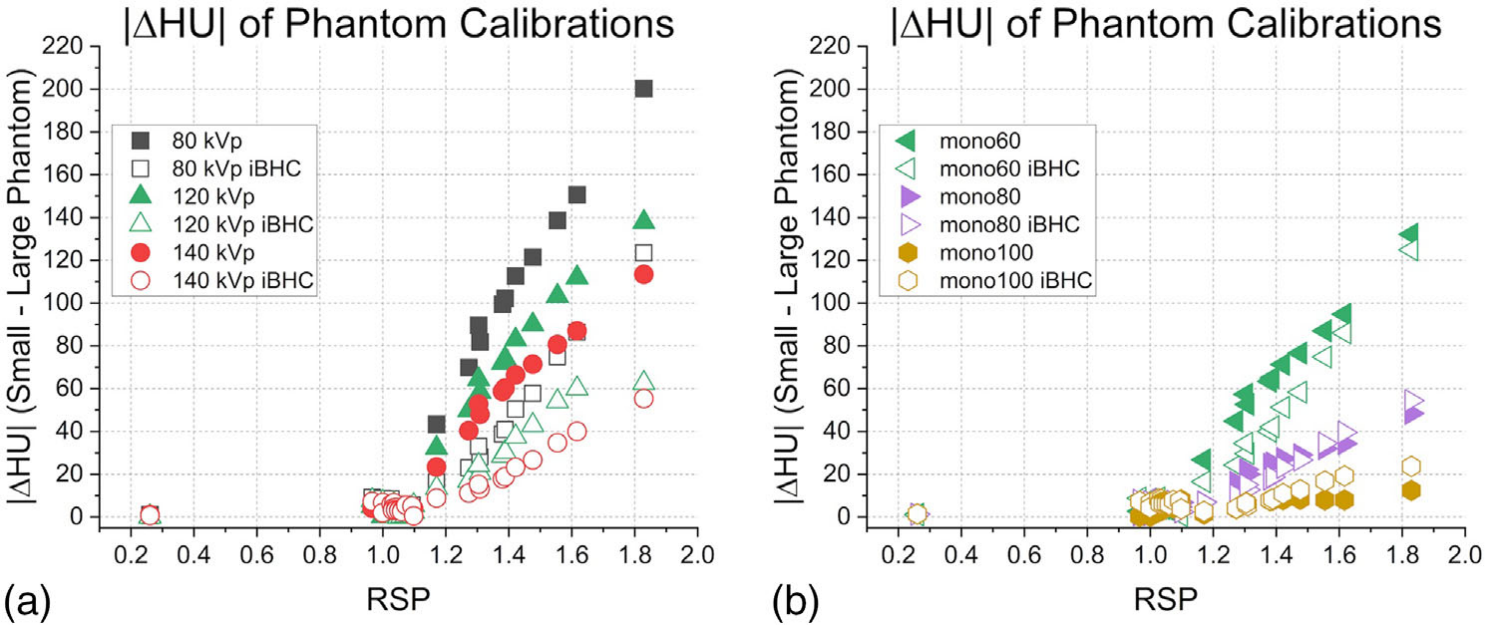
Absolute difference in Hounsfield units (HU) (|ΔHU|) between 20‐cm diameter water/PMMA phantom and Model 467 phantom (33‐cm diameter) at given tissue relative stopping power (RSP) for single‐energy computed tomography (SECT) (a) and dual‐energy computed tomography (DECT)‐derived Mono+ (b) techniques with and without iterative beam‐hardening correction (iBHC) applied

The difference between HU to RSP calibrations generated on two phantoms of different sizes increased as RSP increased. In general, the maximum absolute shift of the calibration curve from the Model 467 phantom to the 20‐cm PMMA phantom was approximately 200 HU for 80 kVp and 137 HU for 60 keV at an RSP of 1.82 (Figure 13). With iBHC applied, these ΔHU were reduced to 124 HU and 125 HU for 80 kVp and 60 keV, respectively. For most soft tissue (RSP < 1.2), the maximum ΔHU was less than 10 between the calibrations.

**FIGURE 14:**
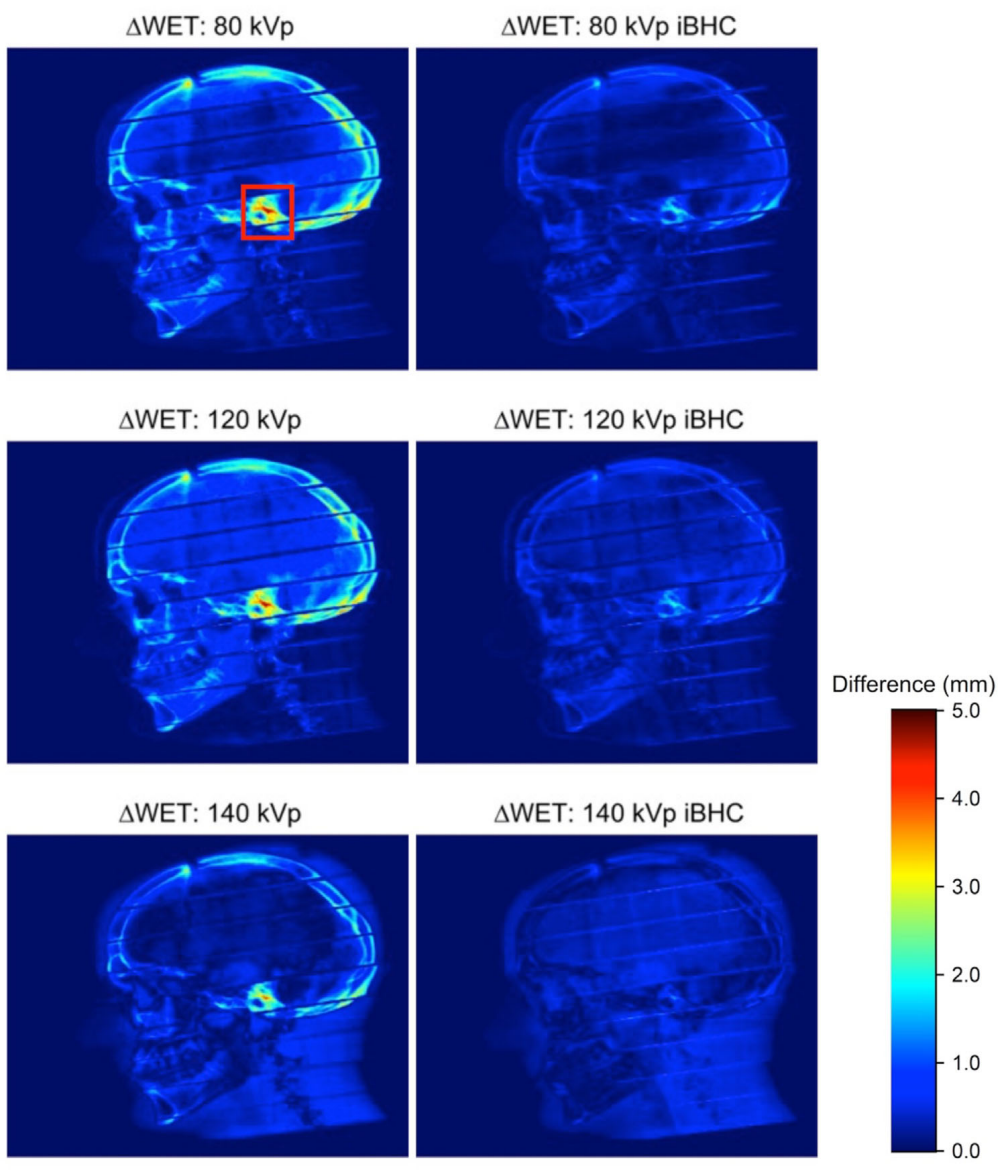
The difference in the water‐equivalent thickness projections (ΔWET) of head anthropomorphic phantom at 90 degrees derived from 20‐cm diameter water/PMMA phantom and Model 467 phantom calibrations with and without iterative beam‐hardening correction (iBHC) applied for single‐energy computed tomography (SECT) techniques. Red outline indicates the area used for the region‐of‐interest (ROI) histogram analysis.

**FIGURE 15:**
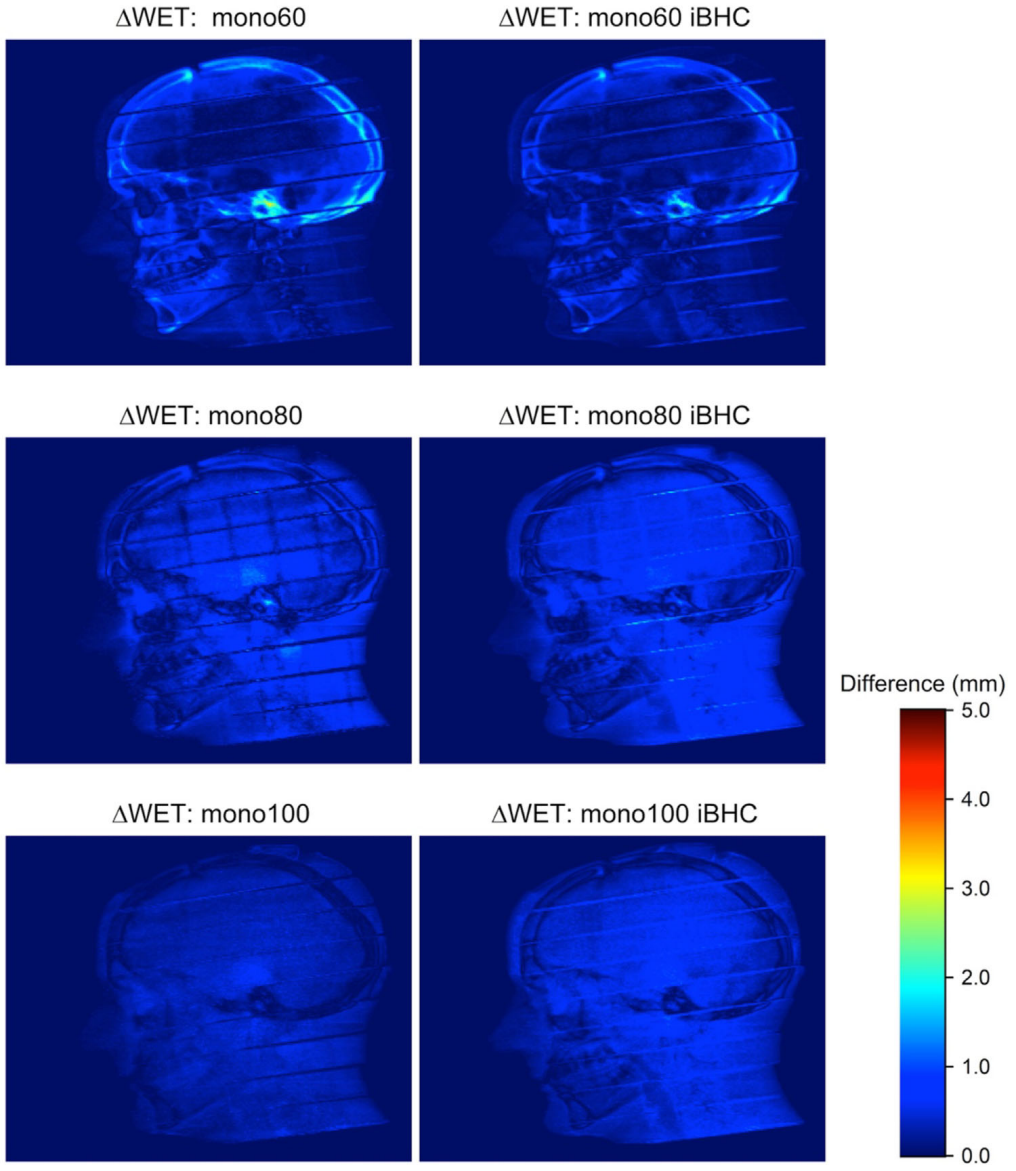
Difference in the water‐equivalent thickness projections (ΔWET) of head anthropomorphic phantom at 90 degrees derived from 20‐cm diameter water/PMMA phantom and Model 467 phantom calibrations with and without iterative beam‐hardening correction (iBHC) applied for dual‐energy computed tomography (DECT)‐derived Mono+ techniques

**FIGURE 16:**
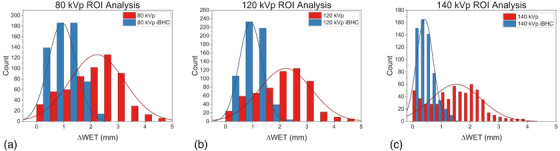
Region‐of‐interest (ROI) histogram analysis of petrous pyramids of the anthropomorphic phantom for single‐energy computed tomography (SECT) techniques at (A) 80 kVp (B) 120 kVp (C) 140 kVp

**FIGURE 17:**
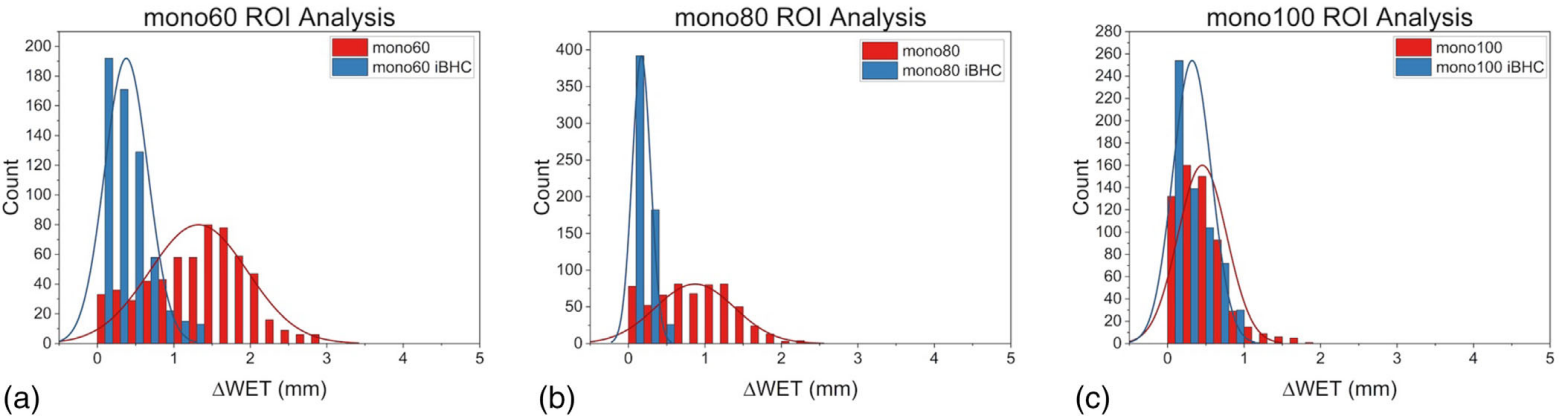
Region‐of‐interest (ROI) histogram analysis of petrous pyramids of the anthropomorphic phantom for dual‐energy computed tomography (DECT)‐derived techniques Mono+ techniques at (A) mono60 keV (B) mono80 keV (C) mono100 keV

When these calibrations were applied to the anthropomorphic phantom, the differences from 90‐degree WET projections were plotted in Figure 14 for SECT and Figure 15 for Mono+. Maximum voxel differences of approximately 5 mm in the WET were observed in the cortical bone regions of the skull. The use of iBHC during calibration reduced these differences. Histogram analysis was done for the selected ROI indicated in Figures 14 and 15 for both subsets of techniques (Figures 16 and 17), and the mean shift of the distributions is summarized in Tables 3 and 4. With the inclusion of iBHC, the ΔWET of the ROI was reduced by 1.3, 1.3, and 1.1 mm for the SECT techniques for the 80, 120, and 140 kVp, and the Mono+ ΔWET was reduced by 0.9, 0.7, and 0.1 mm for the 60, 80, and 100 keV techniques respectively.

**TABLE 3:** Region‐of‐interest (ROI) analysis for mean difference in the water‐equivalent thickness (ΔWET) in the anthropomorphic head phantom ROI for single‐energy‐computed tomography (SECT) techniques

**Technique**	80 kVp	120 kVp	140 kVp	80‐kVp iBHC	120‐kVp iBHC	140‐kVp iBHC
**Mean ΔWET (mm)**	2.2	2.2	1.5	0.9	0.9	0.4

Abbreviation: iBHC, iterative beam‐hardening correction.

**TABLE 4:** Region‐of‐interest (ROI) analysis for mean difference in the water‐equivalent thickness (ΔWET) in the anthropomorphic head phantom ROI for Mono+ techniques

**Technique**	mono60	mono80	mono100	mono60 iBHC	mono80 iBHC	mono100 iBHC
**Mean ΔWET (mm)**	1.3	0.9	0.5	0.4	0.2	0.3

Abbreviation: iBHC, iterative beam‐hardening correction.

## 4 DISCUSSION

A large source of systematic range uncertainty in proton radiotherapy is associated with the underlying CT scans used to infer material parameters from the observed HU. In practice, this uncertainty limits treatment plans that can maximize the dose‐sparing potential of proton radiotherapy, such as exclusive en face beam arrangements. However, estimates of this range uncertainty from CT do not account for artifacts such as BHA.^31^ The varied manifestations of BHA make manual isolation and correction difficult during treatment planning: Cupping may be difficult to identify in a heterogeneous medium such as patient anatomy, and streaking may be diffuse through multiple projection angles. The use of DECT has been proposed for proton radiotherapy to better characterize the material decomposition of calibration materials to patient tissue, as it can account for variations in tissue compositions better than the SECT stoichiometric approach.^32^ Although DECT has well‐established theoretical robustness against BHA as compared to SECT techniques, DECT‐based parameter extraction methods for RSP calculation have shown a paradoxical sensitivity to CT number variation such as from beam hardening.^33^, ^34^ The net benefits in the use of DECT for proton radiotherapy therefore need to be carefully evaluated. In this present work, and we compare DECT‐based monochromatic and parameter extraction techniques against SECT with and without the use of a vendor‐specific beam‐hardening correction algorithm by inducing BHA in controlled phantom scenarios.

The SECT and DECT generated pseudo monochromatic techniques could be compared by calibrating both for HU to RSP with the standard stoichiometric approach. The use of iBHC introduced a systematic shift toward higher HU for a given RSP in the calibration curve for each respective technique (Figure 5). However, the shift in the calibration curve due to iBHC is approximately half of the difference between large and small phantom calibrations (Figure 13). Generating technique‐ or size‐specific calibration curves may be considered part of the overall uncertainty budget in a radiation therapy quality management program in the clinic.^35^ The vendor‐specific parameter extraction (*syngo*.via Rho/Z) has the clear advantage of not requiring a user‐generated HU to RSP calibration, but considerations of the uncertainties of this approach are limited due to its proprietary nature.

Comparing SECT techniques in the low frequency or cupping BHA scenario, increasing the X‐ray tube maximum energy (kVp) results in overall lower dependence of the bone HU and resulting RSP ratio on phantom size (Figures 6 and 7). This is in keeping with the proportionally smaller dependence on the photoelectric effect as the maximum spectrum energy increases and therefore reduced CT beam‐hardening effect. However, when applying the respective calibration curves, the slope of the phantom size to RSP ratio relation was largely the same for all SECT energies. As the spectrum kVp increases, the slope of the calibration curve from spongiosa to cortical bone increases for SECT techniques (Figure 5a) nearly inversely proportionate with the decrease in the phantom size to HU relation (Figure 6a). This suggests that for proton RSP computation, one cannot simply increase the kVp of the CT acquisition to combat beam hardening. The introduction of iBHC greatly reduces this dependence on phantom size for SECT (Figure 6a), indicating substantial mitigation of cupping BHA for SECT evident particularly at *d*_eff_ of 26.9 cm and larger. The baseline shift of the RSP ratio (test RSP to calibration RSP of cortical bone) represents inherent modeling error and associated uncertainty.

The use of DECT‐generated pseudo‐monochromatic images using the Mono+ algorithm resulted in a far weaker effect of phantom size on HU and RSP (Figures 6b and 7c), with near independence at the higher reconstructed energies of 80 and 100 keV. The baseline shift of the RSP ratio was smaller with higher energy reconstructions. The use of iBHC with Mono+ reconstructions reduced the baseline shift of the 60‐keV technique but did not significantly change the phantom size to RSP ratio relationship. Using iBHC with SECT at the kVp values tested resulted in nearly identical robustness against cupping BHA compared with monochromatic reconstructions greater than 80 keV, and a clear advantage at 60 keV.

The *syngo*.via Rho/Z algorithm demonstrated a positive correlation of the RSP ratio with phantom size (Figure 8). The strength of the correlation was weakened with the inclusion of the iBHC algorithm, and an overall reduction in the RSP obtained. Michalak et al. studied the effect of phantom size on RSP error for the Rho/Z algorithm and found a negative correlation for dense bone with respect to phantom size.^36^ However, the data indicated a large drop in RSP error only at a phantom size of 45‐cm diameter, much larger than the Model 467 calibration phantom. Further investigation into the correlation is limited by a lack of published methodology and correction factors of the Rho/Z algorithm.

In the high‐frequency or streaking BHA scenario for SECT techniques, a clear energy dependence can be seen with respect to HU and RSP difference between the artifact and artifact‐free regions of the phantom (Figures 9, 10, 11). Streaking BHA results in lower attenuation for regions adjacent to highly attenuating tissue such as bone. The absolute difference in RSP decreased by approximately 1.8% from 80 to 140 kVp. With the inclusion of iBHC, the effect of streaking BHA was mitigated; at 140 kVp with iBHC, the effect of the BHA on the RSP difference was neutralized. The results of the Mono+ techniques demonstrated that DECT‐based monochromatic imaging is still susceptible to streaking BHA, depending on reconstructed energy, as also demonstrated by Ueguchi et al.^25^ At 100 keV, however, the RSP difference was virtually eliminated. Notably, the inclusion of iBHC with the monochromatic techniques provided dramatic improvement for HU and RSP differences in the presence of streaking BHA. The Rho/Z algorithm resulted in relatively less of RSP difference in this scenario but similarly benefitted from iBHC that brought the RSP difference to zero.

The data from the first two phantom scenarios suggest that iBHC can assist in providing additional robustness of the HU to RSP calibration. Ainsley et al. found that user‐generated CT calibration was relatively robust against variations in numerous factors such as the position of the ROI in the image plane, scan slice thickness, and phantom vendor selection among other variables but concluded that variations in anatomical dimensions may warrant “two or more” calibration curves in practice.^37^ Figures 14 and 15 attempt to quantify the magnitude of the effect of varying calibration phantom size on an anthropomorphic phantom. The ΔWET at a projection angle of 90 degrees derived from the 20 and 33 cm calibration phantoms resulted in a maximum of 5 mm for SECT techniques without iBHC in the petrous portion of the temporal bone. The overall ΔWET in the skull lowered as kVp increased, as did the mean of the ROI (Figure 16). Notable improvement was made with the inclusion of iBHC, to less than 1 mm at 140 kVp. With DECT monochromatic techniques, a maximum ΔWET of 3 mm was obtained at 60 keV but reduced to less than 1 mm at 80 and 100 keV.

In each phantom scenario, the use of iBHC enabled SECT techniques to achieve similar robustness against varying forms of BHA as DECT‐derived monochromatic images. Though DECT can provide additional uncertainty reduction with regard to tissue composition variation, the potential to reduce BHA‐related CT uncertainty with relatively lower cost CT imaging may provide significant benefit to the clinic. The accuracy of dose computation in treatment planning is itself underpinned by HU accuracy. The presence of cupping and streaking BHA shifts HU values in the image dataset lower than reality and has the potential to result in under‐ranging in treatment plan design. Although outside the scope of this study, the use of iBHC to suppress BHA artifacts may also provide image quality benefits to treatment planning in target and organ segmentation.

One limitation of this work is the lack of polychromatic DECT‐based approaches, such as those summarized by Bär et al.^11^ Although established in literature, the lack of TPS vendor support makes clinical application of these calibration methods difficult. In addition, phantom sizes greater than 30.7‐cm *d*_eff_ could not be constructed for the examination of cupping BHA. Therefore, clinical scenarios with patient sizes greater than the calibration phantom size are outside the scope of this study and warrant further investigation. Only Siemens’ algorithms could be evaluated in this work, and the relative robustness against BHA for the specified techniques may vary across each vendor's implementation of artifact reduction software. Finally, it is important to note that the effects of BHA are limited outside of the bone tissue region. The relative differences described in this study therefore cannot be directly applied to the patient scenario, as individual patient anatomy comprises varied tissue types.

## 5 CONCLUSIONS

The two distinct manifestations of CT BHA can result in characteristically different effects when applied to proton RSP computation, depending on scan technique. The use of beam‐hardening correction software (iBHC) improved RSP computation in all tested scenarios and increased the robustness of user‐generated calibrations against variations in subject size. Finally, iBHC provided parity between certain SECT and DECT techniques in relative robustness against BHA.

## AUTHOR CONTRIBUTION

Michael S. Chacko designed and carried out experiments. Hardev S. Grewal helped review results. Dee Wu and Jagadeesh R. Sonnad reviewed all results and manuscript. Michael S. Chacko wrote the manuscript with input from all authors.

## CONFLICT OF INTEREST

None to report.

## Data Availability

The data that support the findings of this study are available from the corresponding author upon reasonable request.
